# Functional consequences of copy number variants in miscarriage

**DOI:** 10.1186/s13039-015-0109-8

**Published:** 2015-01-31

**Authors:** Jiadi Wen, Courtney W Hanna, Sally Martell, Peter CK Leung, Suzanne ME Lewis, Wendy P Robinson, Mary D Stephenson, Evica Rajcan-Separovic

**Affiliations:** Department of Pathology and Laboratory Medicine, University of British Columbia, Vancouver, V6T 2B5 Canada; Child & Family Research Institute, Vancouver, V5Z 4H4 Canada; Department of Medical Genetics, University of British Columbia, Vancouver, V6T 1Z3 Canada; Department of Obstetrics and Gynaecology, University of British Columbia, Vancouver, V6Z 2 K5 Canada; Department of Obstetrics and Gynecology, University of Illinois at Chicago, Chicago, 60612 USA

**Keywords:** Miscarriage, Copy number variation, *TIMP2*, *OFD1*, *TRAPPC2*, Gene expression

## Abstract

**Background:**

The presence of unique copy number variations (CNVs) in miscarriages suggests that their integral genes have a role in maintaining early pregnancy. In our previous work, we identified 19 unique CNVs in ~40% of studied euploid miscarriages, which were predominantly familial in origin. In our current work, we assessed their relevance to miscarriage by expression analysis of 14 genes integral to CNVs in available miscarriage chorionic villi. As familial CNVs could cause miscarriage due to imprinting effect, we investigated the allelic expression of one of the genes (*TIMP2)* previously suggested to be maternally expressed in placenta and involved in placental remodelling and embryo development.

**Results:**

Six out of fourteen genes had detectable expression in villi and for three genes the RNA and protein expression was altered due to maternal CNVs. These genes were integral to duplication on Xp22.2 (*TRAPPC2* and *OFD1)* or disrupted by a duplication mapping to 17q25.3 (*TIMP2*). RNA and protein expression was increased for *TRAPPC2* and *OFD1* and reduced for *TIMP2* in carrier miscarriages. The three genes have roles in processes important for pregnancy development such as extracellular matrix homeostasis *(TIMP2 and TRAPPC2)* and cilia function *(OFD1). TIMP2* allelic expression was not affected by the CNV in miscarriages in comparison to control elective terminations.

**Conclusion:**

We propose that functional studies of CNVs could help determine if and how the miscarriage CNVs affect the expression of integral genes. In case of parental CNVs, assessment of the function of their integral genes in parental reproductive tissues should be also considered in the future, especially if they affect processes relevant for pregnancy development and support.

**Electronic supplementary material:**

The online version of this article (doi:10.1186/s13039-015-0109-8) contains supplementary material, which is available to authorized users.

## Background

Genetic factors, such as single gene defects and chromosomal abnormalities, are a common cause of miscarriage [1-3]. Their identification is important for informed reproductive decisions and counselling and is the key goal of reproductive genetics [4,5]. The detection of chromosomal abnormalities has been recently revolutionized with the development of chromosome microarray analysis (CMA) which facilitates the detection of large scale and subtle chromosomal microdeletions and microduplications (DNA copy number variants or CNVs). CMA is based on the assessment of DNA directly obtained from tissues and thus minimizes the negative impact of tissue culture artefacts and failure associated with traditional cytogenetic analysis of miscarriages.

Due to its many benefits, including improved resolution and diagnostic yield, CMA is now considered a first line test for subjects with postnatal developmental delay and congenital abnormalities [6,7]. It identifies clinically relevant CNVs in 10-15% of chromosomally normal cases, with the majority of CNVs undetectable by routine karyotyping. In contrast, array studies of miscarriages are still rare, and no more than 3000 cases worldwide were reported [3]. Most of the miscarriage cases (2392) were described in the recent publication by Levy et al. [8], which reported putatively causative CNVs in 1.6% of chromosomally normal cases. These CNVs were defined as >5 Mb or overlapping with critical regions causing or predisposing to viable microdeletion/duplication syndromes.

Interestingly, the majority of CNVs identified so far in miscarriages with a normal karyotype were familial in origin. CNVs inherited from normal parents are generally considered less likely to be causative of an abnormal phenotype, however, they can be pathogenic if they i) uncover a mutation within the intact allele of the developmental gene in the conceptus, ii) contain genes with variable expressivity or penetrance iii) involve imprinting (parent of origin dependent) genes. In addition, parental CNVs could still lead to miscarriage if they affect genes required for normal parental reproductive function, including, for example, genes required for placenta function, maternal preparation for and maintenance of pregnancy and genomic stability of the sperm. These processes are essential for successful pregnancy outcome [9], but may not necessarily impair parents’ overall health status and can demonstrate pathogenic characteristics only at the time of pregnancy. Recently, Nagirnaja et al. [10], suggested that a CNV from 5p13.3 chromosomal region is enriched in women with recurrent pregnancy loss (RPL) in comparison to fertile controls and could represent a risk factor for pregnancy complications, as it contains genes predominantly expressed in placenta.

We have recently performed CMA analysis of miscarriages from couples with RPL and sporadic miscarriages with embryoscopy findings [11,12]. Unique CNVs not reported in Database of Genomic Variants were noted in ~40% of miscarriages and were predominantly parental in origin, making their interpretation more challenging. In order to further investigate the potential role of these CNVs in miscarriage we performed functional analysis (RNA and protein expression) of genes integral to miscarriage CNVs, using chorionic villi from miscarriages from 6 families with RPL, reported in our previous work. Our study shows changes in RNA/protein expression for 3/14 tested genes from maternal CNVs, which could be of relevance for miscarriage due to their role in processes important for growth of the conceptus and/or maternal preparation for and support of pregnancy. Functional studies of miscarriage CNVs could therefore help identify miscarriage candidate genes, but should be accompanied with functional studies of CNVs in reproductive tissues of carrier parents.

## Results

### Expression of genes integral to CNVs in miscarriage cell cultures

**Figure 1:**
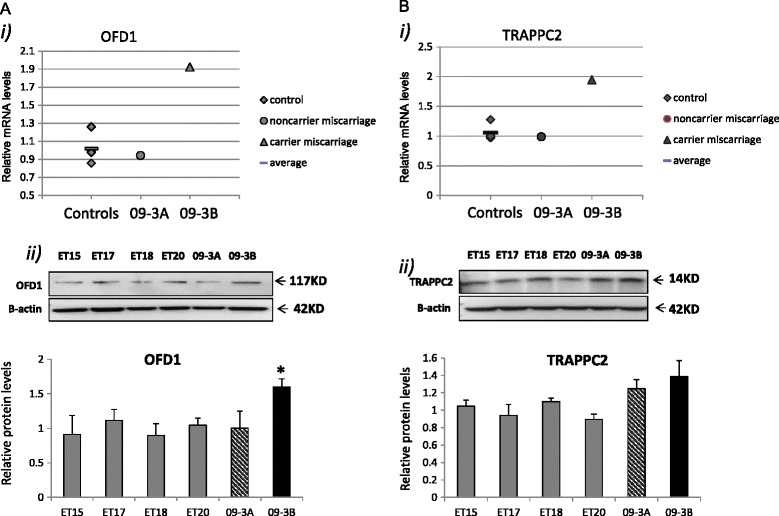
**mRNA and protein expression of OFD1 and TRAPPC2 in primary culture of human chorionic villi.** ET 15, 17, 18 and 20 indicate control samples from the cell cultures of elective terminations (n = 4). 09-3A and B indicate miscarriages from female 09–1. mRNA (i) and protein (ii) values for **(A)** OFD1 and **(B)** TRAPPC2 expression were normalized to the corresponding ß-actin mRNA levels. The results derived from at least three repeats of each sample and the mean level of each sample is represented in the scatter graph. The difference between the four control samples (as a group) and the two CNV carrier miscarriages, as two groups, has been evaluated by Student t-test (*, p < 0.05).

**Figure 2:**
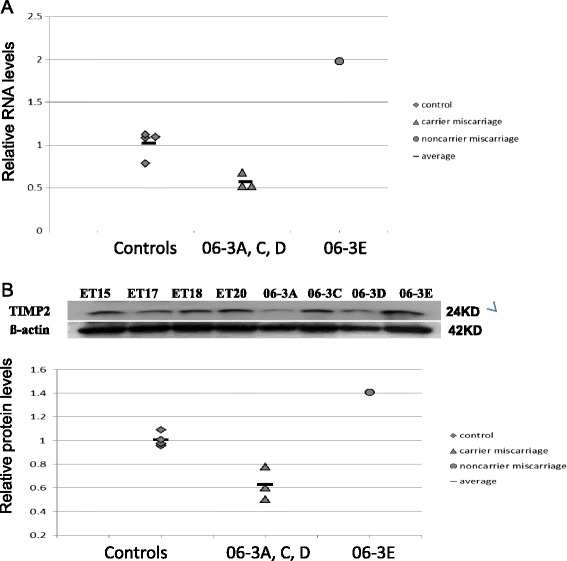
**mRNA and protein expression of** ***TIMP2*** **in primary culture of human chorionic villi.** ET15, 17, 18 and 20 indicate control cell cultures from elective terminations (n = 4). 06-3A, C, D and E indicate miscarriages of the female 06–1 (n = 4). **(A)** mRNA and **(B)** protein values for *TIMP2* expression were normalized to the corresponding ß-actin mRNA levels. The results derived from at least three repeats of each sample and the mean level of each sample is represented in the scatter graph. The difference between the four control samples and the three CNV carrier miscarriages, as two groups, has been evaluated by student t-test (*, p < 0.05).

Three genes, *OFD1, TRAPPC2* and *TIMP2* out of 14 selected for expression analysis had altered mRNA and protein expression in cultured miscarriage chorionic villi cells (Table 1 and Figures 1 and 2). For *NDUFAF2, CHSY3* and *PRMT3* (Table 1) the expression in control pregnancies and miscarriages with CNVs was comparable. The remaining genes assayed had either very low or no expression in cultured chorionic villi from controls (*PARK, LIPC, CTNNA3, EGFL6, GPM6B, RAB9A, POU6F2* and *C7orf10*) and were not assessed in miscarriages.

**Table 1:** **CNV gene analysis**

**Samples**	**Miscarriage**	**Locus**	**Breakpoint**	**CNV size (kb)**	**Type of CNV and origin**	**Gene picked for expression (type of CNV abnormality)**	**mRNA expression in control elective terminations/miscarriage that carries the CNV**	**Protein expression**
**Miscarriages from reference Rajcan-Separovic et al.** [12]	03-3A	6q26	162126633-162271770	145	loss-pat	PARK2 (loss involves part of exon and intron)	N/not attempted	
	05-3A	5q12.1	60407026-60464658	58	loss-pat	NDUFAF2 (loss involves exon 3)	yes/no difference between CNV carriers and controls	
	06-3A,B,C and D (not in 3E)	17q25.3	74381287-74466887	86	gain-mat	TIMP2 (gain involves exon1,2)	yes/decrease in CNV carriers	decreased
	06-3E	15q22.1	56487120-56562873	76	loss-pat	LIPC (loss of exon1)	N/not attempted	
	07-3A	10q21.3	67992425-68064617	72	loss-mat	CTNNA3 (loss involves exon11)	N/not attempted	
	09-3B (not in 3A)	Xp22.2	13415099-13745233	33	gain-mat	EGFL6 (complete gain)	N/not attempted	
						GPM6B (almost complete gain)	N/not attempted	
						OFD1 (complete gain)	yes/increase in CNV carrier	increased in CNV carrier
						RAB9A (complete gain)	N/not attempted	
						TRAPPC2 (complete gain)	yes/increase in CNV carrier	increased in CNV carrier
	10-3A	5q23.3	129388119-129441487	53	loss-pat	CHSY3 (CSS3) (loss involes intron)	yes/no difference between CNV carrier and controls	
		11p15.1	20442396-20559837	117	Gain-pat	PRMT3 (gain involves exon9-11)	yes/no difference between CNV carrier and controls	
		7p14.1	39470588-39647671	177	Gain-mat	POU6F2 (gain involves last exon)	N/not attempted	
						C7orf10 (complete gain)	N/not attempted	

N = expression in control villi tissue low or not detectable, the expression in miscarriage therefore not attempted.

*OFD1* and *TRAPPC2* are integral to a CNV on Xp22.2 (duplication with breakpoints from 13415099–13745233) identified by Agilent 105 k array (Figure One in Rajcan-Separovic et al. [12]) in female 09–1 who had 6 miscarriages (current paper, Additional file 1: Table S1). Two of her 6 miscarriages had array analysis: male miscarriage 09-3A had a normal array result, while female miscarriage 09-3B inherited the maternal Xp22.2 CNV. These two miscarriages were available for functional analysis. Increased RNA and protein expression for *OFD1* and *TRAPPC2* was detected in 09-3B (Figure 1), while the remainder of the genes from the Xp22.2 CNV (*EGFL6, GPM6B*, and *RAB9A*) had low or undetectable expression in control ET cell cultures and were not evaluated in 09-3B (Table 1). Random chromosome X inactivation was identified in 09-3B, while the mother’s chromosome X inactivation status was uninformative [12].

The CNV disrupting the *TIMP2* gene mapped to 17q25.3 (duplication with breakpoints from 74,381,287 -74,466,887) and was detected in female 6–1 and in 4/5 available miscarriages, as reported previously (Figure Two in Rajcan-Separovic et al. [12]). Cell cultures from chorionic villi were available from four miscarriages (06-3A, C, D which contained the CNV and 06-3E which did not). The three miscarriages with the *TIMP2* CNV (06-3A, C and D) showed a ~50% decrease in mRNA and protein expression in comparison to four control elective terminations (ET 15, 17, 18 and 20). A ~2-fold increase of *TIMP2* mRNA and protein was noted in the fourth miscarriage (06-3E), which did not carry the *TIMP2* CNV (Figure 2).

### TIMP2 Allelic expression analysis

**Figure 3:**
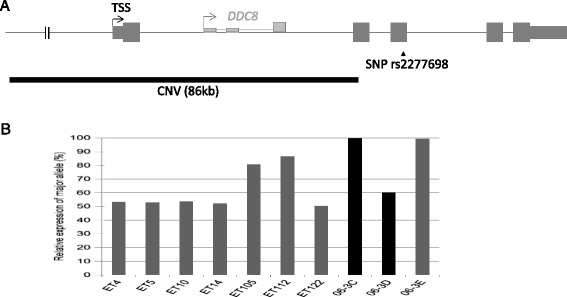
**Allelic expression of** ***TIMP2*** **gene. (A)** schematic diagram of the structure of the *TIMP2* gene region, including its 5 exons (grey bars) and transcription start site (TSS). Important genomic features, including the copy number variation (CNV) identified in the recurrent miscarriage family, and a polymorphic coding single nucleotide polymorphism (SNP) used to assess *TIMP2* allelic expression have been labelled. **(B)** Percentage gene expression of the major allele for rs2277698 in exon 3 of *TIMP2* gene in informative chorionic villi from 1st trimester elective terminations (ET) and miscarriages from recurrent miscarriage family 06. Miscarriage 06-3C and 06-3D had the CNV while miscarriage 06-3E did not.

Based on previous reports suggesting preferential maternal expression of *TIMP2* in placenta [13], we tested the parent-of-origin specific expression of *TIMP2* in control ET and in miscarriages from female 06–1 to determine if the CNV affected the *TIMP2* allelic expression. Monoallelic expression of the maternal allele was detected in 2 of 3 informative miscarriages (06-3C with and 06-3E without the CNV, Figure 3) while a close to biallelic expression (~60%) was noted in the third miscarriage (06-3D) which was trisomic for chromosome 16 and contained the *TIMP2* CNV. Monoallelic expression of the maternal allele was also noted in two of the seven informative ET samples heterozygous for the polymorphic rs2277698 G/A SNP in exon 3 (out of 35 genotyped). The cells from the remaining 5 control and informative ETs had biparental expression.

## Discussion

In this study we explored further the miscarriage CNVs we identified in our previous work by testing the expression levels of their integral genes in available miscarriage tissues with CNVs. Three genes integral to maternal CNVs had altered expression in miscarriages consistent with genomic alternation caused by the CNV.

*TRAPPC2* and *OFD1* had increased RNA and protein expression in miscarriage 9-3B with a gain of Xp22.2. *TRAPPC2* has a role in procollagen transportation [14] while *OFD1* regulates cilia function [15]. Collagens are the main components of extracellular matrix (ECM) and provide structural support for the tissues, but also play important roles in cell growth, differentiation, adhesion, and migration [16,17]. The dysfunction of *OFD1*, through abnormal ciliogenesis, results in defects in sonic hedgehog (Shh) and canonical Wnt signaling pathways [15,18-20] which are linked to abnormal implantation and embryonic development [21-23]. Although the mis-expression of *TRAPPC2* and *OFD1* could potentially explain miscarriage 9-3B, the Xp22.2 CNV was not present in miscarriage 09-3A, and therefore cannot be associated with all the pregnancy losses of female 09–1. Instead, the unifying cause of her miscarriages could be the effect of the CNV on maternal *OFD1* and *TRAPPC2* gene expression, particularly because of the reports of abnormal uterine collagen content resulting in pregnancy loss [24,25]. It would be of interest the explore the expression of these genes in the decidua of female 9–1. Unfortunately this tissue was not available for analysis.

*TIMP2* is the third gene that showed altered expression in miscarriages and is known to inhibit matrix metaloproteases (MMPs), which degrade extra cellular matrix (ECM), and have a critical role in tissue remodelling and angiogenesis in placenta/endometrium [26-29]. Expression of *TIMP2* was reduced in miscarriages with the CNV, possibly because the CNV (duplication) disrupts the 5′ end of the gene and causes structural alternations in the genomic region of *TIMP2*. It has been previously observed that the genomic position of regulatory elements in the *TIMP2* promoter (AP-1) affects the gene expression [30]. However, the CNV appears not to be the sole cause of recurrent miscarriage in this family as TIMP2 expression was also altered (increased) in the miscarriage without the CNV. It is conceivable that, due to the CNV, the expression of *TIMP2* is also abnormal (reduced) in the endometrium/decidua of female 6–1 and could impair pregnancy development. This is of interest considering that decidual *TIMP2* has a role in regulating trophoblast invasion by modulating trophoblast *MMP* and *TIMP2* expression [31], and in particular inhibiting trophoblast *TIMP2* expression. In keeping with the possibility of the effect of the maternal and/or pregnancy CNV on *TIMP2* function are the pathology findings for six out of ten miscarriages from female 6–1 which demonstrated morphologic abnormalities of the maternal vasculature (intimal hyperplasia of maternal vessels) and/or placenta (perivillous fibrin deposits) [12] (Additional file 1: Table S1).

Familial CNVs are frequently hypothesised to cause an adverse outcome of the pregnancy if they contain or disrupt imprinting genes. Previous reports suggested that *TIMP2* is an imprinted gene with preferential maternal expression in placenta. This was based on observations of reduced expression in placenta from complete moles [13], and overexpression of the maternal copy in a mouse model of RPL [32]. We were therefore interested in finding out if the maternal CNV, disrupting the *TIMP2* gene, affects its allelic expression in miscarriages that carry the CNV. Our parent of origin expression analysis demonstrated biallelic expression in most cases including one miscarriage with the CNV. However, it appears that the regulation of *TIMP2* allelic expression is complex, as it was preferentially maternal in 2/3 miscarriages and 2/7 ETs. It is possible that the allelic expression of this gene is affected by the degree of clonality after placental cell culture, the ratio of methylation of CpG islands (Chernov, et al. [33]), or additional genetic polymorphisms affecting gene expression on one or the other copy. Comprehensive analysis of epigenetic marks at the *TIMP2* gene promoter region(s) in relation to expression is needed to elucidate whether this gene is indeed imprinted and which modifications are important for regulation of gene expression.

## Conclusion

Overall, our findings underscore the need for additional functional characterization of miscarriage CNVs to develop an understanding of the effect of their integral genes on pregnancy development. These studies can be challenging due to lack of miscarriage cell cultures or RNA/protein for functional studies in miscarriages and lack of parental reproductive tissues in cases with parental CNVs. Collection of reproductive tissues (e.g. maternal decidua) for functional analysis would be desirable in future miscarriage CNV analysis. As more miscarriage associated CNVs and genes are identified, their individual and collective role in miscarriage will become more apparent.

## Methods

### Subjects

#### Control pregnancy and miscarriage tissues

Control chorionic villus samples were obtained from first trimester elective terminations (ET) of pregnancy for social reasons by dilation and curettage (6–12 weeks of gestation). The control ET had no evidence of aneuploidy as determined by multiplex ligation dependent probe amplification (MLPA) [34]. Four ET tissues were used for tissue culture, as previously described [35]. Thirty five uncultured ET tissues were assessed for allelic expression analysis of *TIMP2* gene (see below).

Chorionic villus cell cultures of 10 miscarriages from 6 families (03,05,06,07,09 and 10 as described in Table 1 in the current paper and in Rajcan-Separovic et al*.* [12]) were available for RNA and protein expression analysis.Culture conditions were as for the ET tissues [35].

Maternal contamination has been ruled out in all ET and miscarriage cultures by examining microsatellite markers, using standard protocols [12,36]. The use of parental blood samples, control and miscarriage tissues was approved by the Committee for Ethical Review of Research involving Human Subjects, University of British Columbia and Institutional Review Board of the University of Chicago. All subjects gave written informed consent for these studies.

### Expression analysis

#### RNA, DNA and protein extraction

Total RNA, DNA and protein were extracted from the chorionic villus (CV) cell cultures simultaneously using commercially available kits (AllPrep DNA/RNA Mini kit, Qiagen) according to the manufacturer’s instructions. The purity and concentration of total RNA, DNA and protein present in each of these extracts were quantified using a NanoDrop 1000 Spectrophotometer (Thermo Scientific, Wilmington, USA).

For allelic expression analysis, RNA and DNA were extracted from ET tissue stored in RNA later. Genomic DNA (gDNA) was used to obtain fetal genotype and informative (heterozygous) samples were used for the allelic expression analysis.

#### cDNA synthesis

Aliquots of the total RNA extracts (~500 ng) prepared from the CV cell cultures were subsequently reverse-transcribed into cDNA using GeneAmp Gold RNA PCR Core Kit (Applied Biosystems, Melbourne, Australia). High Capacity cDNA Reverse Transcription Kit (Applied Biosystems, Melbourne, Australia) was used to generate cDNA from 35 ET tissues for *TIMP2* allelic expression analysis.

#### Real-time quantitative (q)PCR

RNA expression analysis was performed in cultured chorionic villi from miscarriages with CNVs and four ETs. Fourteen genes integral to the miscarriage CNVs were selected, and bioinformatics tools and publicly available human genome databases (Ensemble Genome Browser, UCSC) are used to select appropriate primers. The genes for expression analysis were selected based on the availability of cells/RNA/protein from miscarriages that contained their copy number change and included: *PARK2, NDUFAF2, TIMP2, LIPC, CTNNA3, EGFL6, GPM6B, OFD1, RAB9A, TRAPPC2, CHSY3, PRMT3, POU6F2,* and *C7orf10* (Table 1). The nucleotide sequences for primers specific for the 14 genes or the housekeeping gene *β-actin* were designed using Primer Express software (Perkin-Elmer Applied Biosystems) and purchased from Integrated DNA Technologies (IDT). Primer sequences for tested regions are listed in Additional file 1: Table S2. The first-strand cDNA generated from the chorionic villi cells served as a template for qRT-PCR using the ABI PRISM 7300 Sequence Detection System (Perkin-Elmer Applied Biosystems) equipped with a 96-well optical reaction plate for primers specific for 14 selected genes and the housekeeping gene, *β-actin*. Real-time qPCR was performed as previously described [37].

#### Western blot analysis

We detected changes in RNA expression for 3 genes in miscarriages: *OFD1* and *TRAPPC2* from Xp22 CNV (present in female 09–1 and one out of two miscarriages) and *TIMP2* from 17q25 CNV (present in female 6–1 and 4/5 miscarriages). Protein expression analysis for these genes was performed using polyclonal antibodies directed against human OFD1 (Abcam, No. ab97861, ON, Canada), TRAPPC2 (generous gift from Dr De Matteis, Italy) and TIMP2 (NovusBiologicals, No. NB100-92000, Littleton, US). To standardize the amounts of protein loaded into each lane, the blots were reprobed with a monoclonal antibody directed against human β-actin (Novus Biologicals, Littleton, US). The ECL Western Blotting system was used to detect the amount of each antibody bound to antigen and the resultant photographic films were analyzed by UV densitometry (GE Healthcare Life Sciences, Pittsburgh, USA). The absorbance values obtained for OFD1, TRAPPC2 or TIMP2 were then normalized relative to the corresponding β-actin absorbance value. The average of OFD1, TRAPPC2 and *TIMP2* protein expression were obtained from 3 independent replicates for each miscarriage and ET control samples.

#### Allelic expression analysis of TIMP2 gene

We assessed the effect of the CNV on allelic expression in miscarriages from female 6–1 rather than methylation since the *TIMP2* promoter is unmethylated in cervix and blood [38], human term placenta (unpublished data) and mouse placenta throughout pregnancy [39]. Three heterozygous SNPs were identified within the exons of the *TIMP2* gene using UCSC Genome Browser: rs7503726 and rs2277698 in the 5′UTR and rs2277698 within exon 3. Genotyping assays were designed for all three SNPs, using the PSQ Assay Design Software; with successful targeted amplification for only the rs2277698 G/A SNP in exon 3 (heterozygosity = 0.241 ± 0.250) (Additional file 1: Table S3). Genotyping was done on the Pyromark MD machine using the PyroGold SQA reagent kit (Qiagen, Hilden, Germany), using gDNA from placenta to obtain fetal genotypes. Genotyping was performed on DNA from 35 ET samples and 5 miscarriages from female 6–1 with RPL, 4/5 carried the *TIMP2* CNV. 7/35 ET and 3/5 tested miscarriages were informative (heterozygous). Parental origin of the rs2277698 alleles in ET and miscarriage samples was determined by assessing maternal genotypes in DNA extracted from the placental decidua or blood. The sequencing primer used for the gDNA assay (Additional file 1: Table S3), was then used to assess whether there was allelic bias in expression of *TIMP2* in the cDNA, after cDNA-specific amplification. The relative percentage of expressed alleles was obtained using the allelic quantification setting on the Pyromark MD software, and averaging of two independent replicates for each sample.

### Statistical analysis

All statistical tests were performed using the VassarStats: Statistical Computation Web Site (Vassar College, Poughkeepsie, USA), R Statistical Software 2.12.0 (The R Project for Statistical Computing, Auckland, New Zealand) or GraphPad Prism 4 computer software (GraphPad, San Diego, CA). Student’s t-test was used to determine significant differences in transcript levels between the four miscarriages from family 6 with available cultures (3 that carry the *TIMP2* CNV and one without it) and four control ETs. p < 0.05 was considered significant. Significance between the variances of the two groups was determined using F- test.

The absorbance values obtained from the real-time qPCR products and the photograph generated by Western blotting were subjected to statistical analysis using GraphPad Prism 4 computer software (GraphPad, San Diego, CA). Statistical significance between the absorbance values were assessed by the analysis of variance (ANOVA), with p < 0.05 considered significant. Comparison of the means between patients and controls were determined using Dunnett’s test. The results are presented as the mean ± S.E.M. from at least three independent experiments.

## Additional file

Additional file 1: Table S1. Pathology description of the recurrent miscarriages with familial CNV. **Table S2.** Primer sequences for Real-time qPCR. **Table S3.** Primers used to assess allelic expression of TIMP2.
