# Supplementary material for: Functional consequences of copy number variants in miscarriage
Source: Mol Cytogenet. 2015 Jan 31;8:6. doi: 10.1186/s13039-015-0109-8 (PMC4324423; doi:10.1186/s13039-015-0109-8)
Supplement: Additional file 1: Table S1. — Pathology description of the recurrent miscarriages with familial CNV. Table S2. Primer sequences for Real-time qPCR. Table S3. Primers used to assess allelic expression of TIMP2. [file 13039_2015_109_MOESM1_ESM.pptx]

## Slide 1
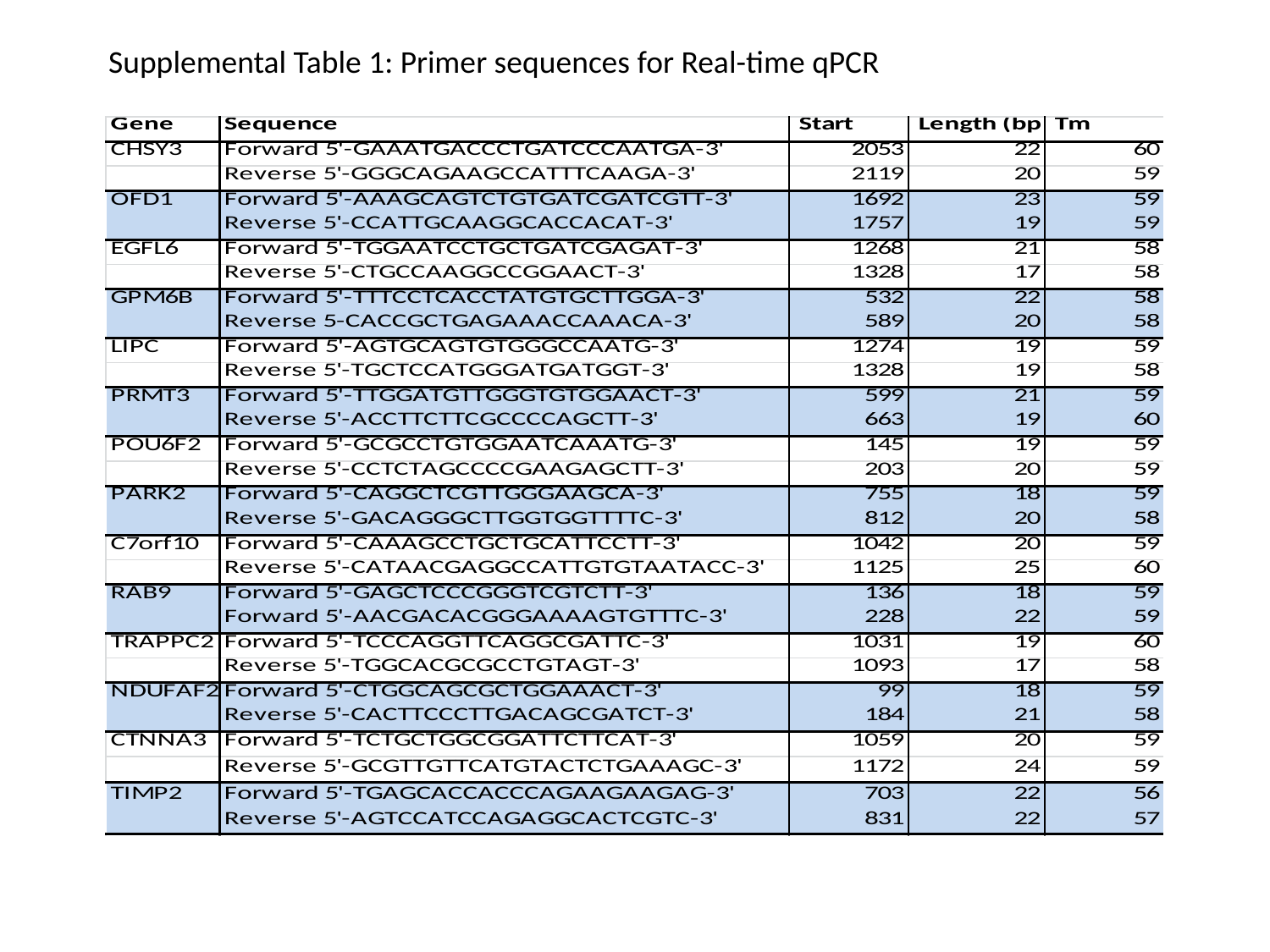

Supplemental Table 1: Primer sequences for Real-time qPCR

## Slide 2
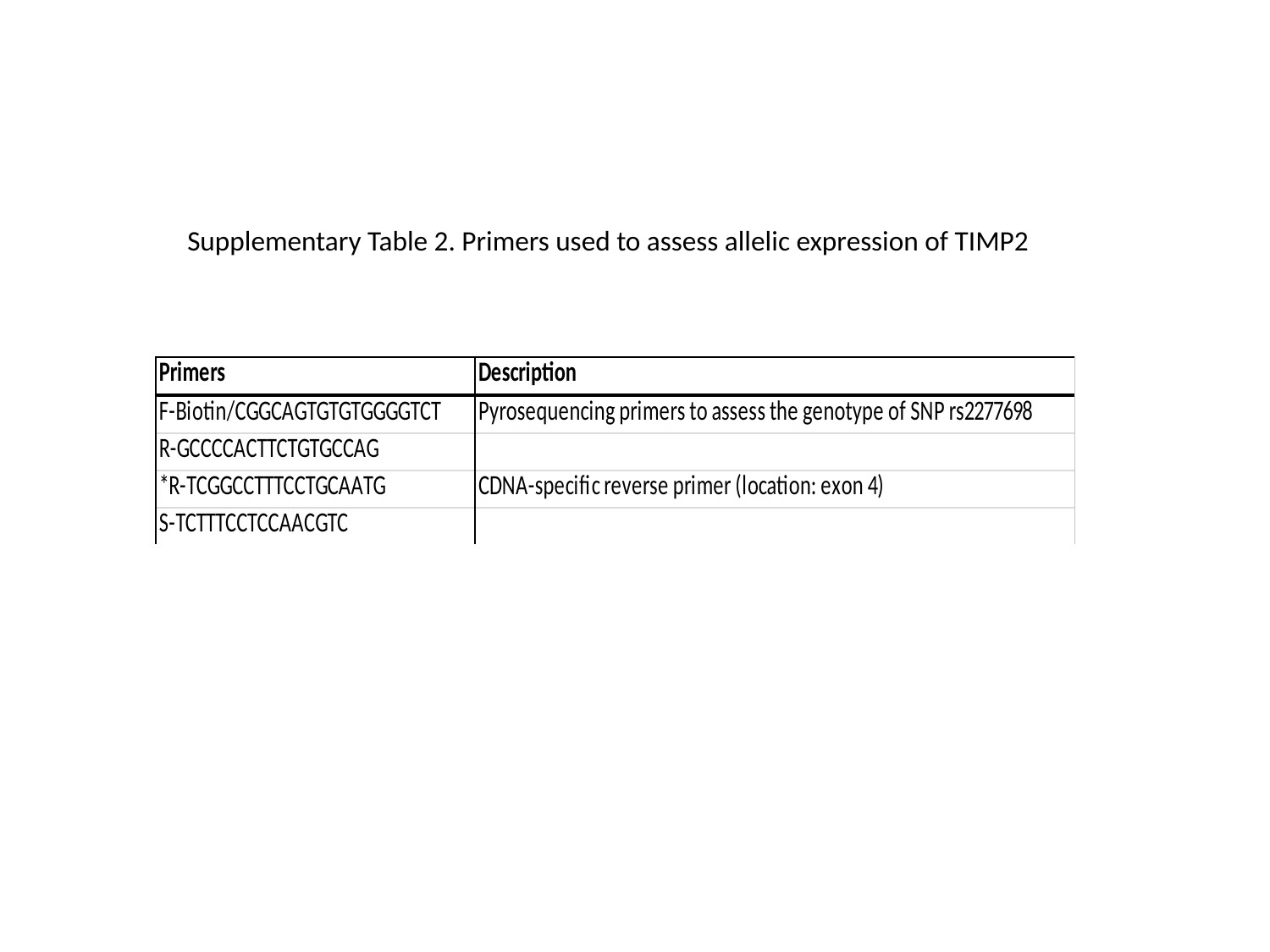

Supplementary Table 2. Primers used to assess allelic expression of TIMP2

## Slide 3
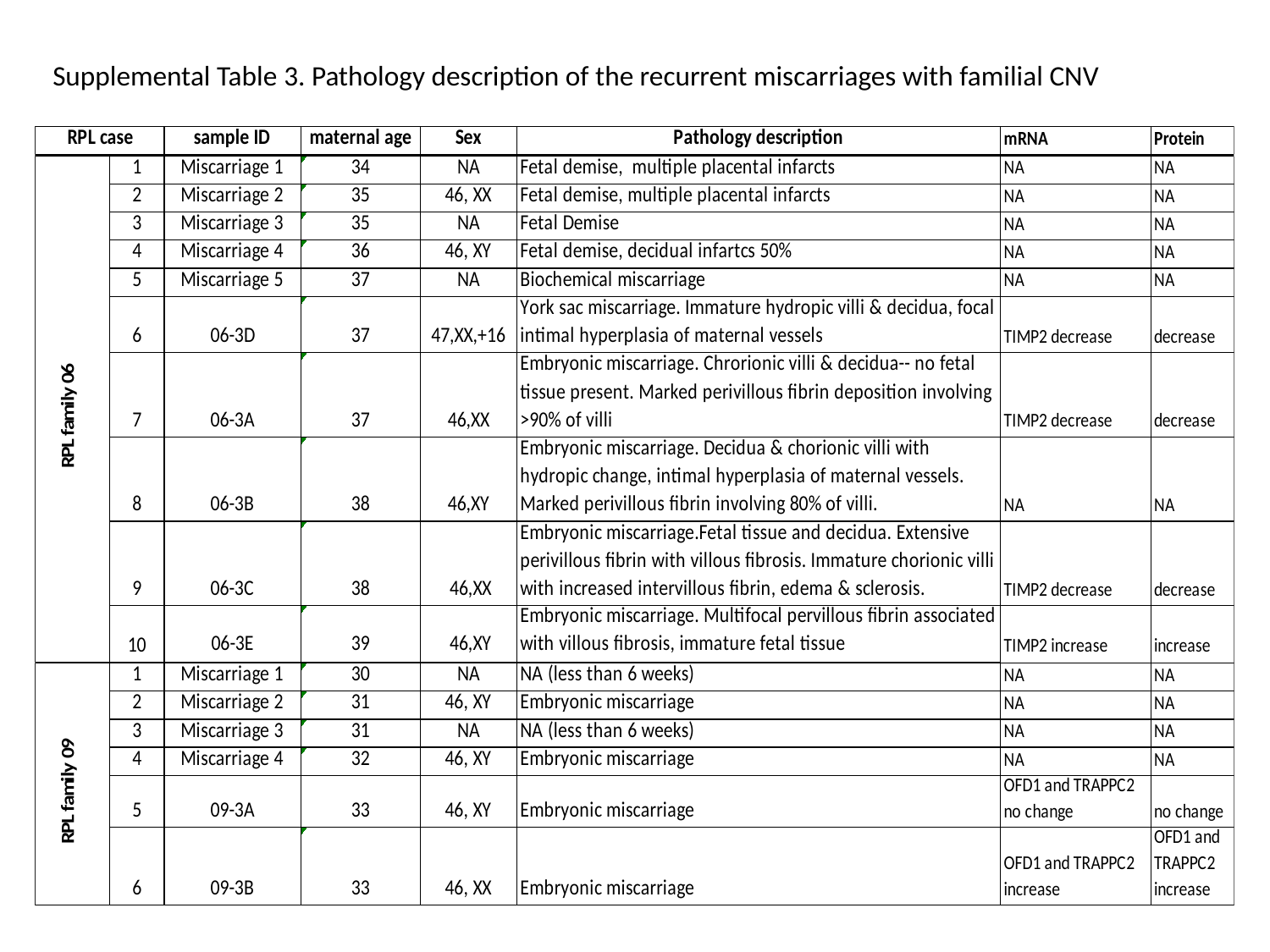

Supplemental Table 3. Pathology description of the recurrent miscarriages with familial CNV
